# A Median Artery of the Corpus Callosum

**DOI:** 10.7759/cureus.3355

**Published:** 2018-09-25

**Authors:** Juan J Altafulla, Emily A Simonds, Graham Dupont, Stefan Lachkar, Zachary Litvack, Joe Iwanaga, R. Shane Tubbs

**Affiliations:** 1 Neurological Surgery, Seattle Science Foundation, Seattle, USA; 2 Miscellaneous, Seattle Science Foundation, Seattle, USA; 3 Neurological Surgery, Seattle Science Foundation, Seattle , USA; 4 Clinical Anatomy, Seattle Science Foundation, Seattle, USA; 5 Neurosurgery, Swedish Neuroscience Institute, Seattle, USA; 6 Medical Education and Simulation, Seattle Science Foundation, Seattle, USA; 7 Neurosurgery, Seattle Science Foundation, Seattle, USA

**Keywords:** anterior communicating artery, endovascular, cerebrovascular, anatomical variant, median artery of corpus callosum

## Abstract

The anterior communicating artery is one of the main components of the vascular network that delivers blood to the brain. Therefore, a good understanding of the normal anatomy and its variations is important to neurologists, neurosurgeons, and other health care providers dealing with the central nervous system. Here, we present a case of a median artery of the corpus callosum found in a cadaver, with consideration of cerebral hemodynamics implications.

## Introduction

Proper knowledge of the variations of the anterior communicating artery (AComA) is fundamental in a clinical setting, as it serves as one of the components of the circle of Willis in regulating, stabilizing, and balancing the anterior cerebral circulation [1].

The AComA acts as a bridge between the left and right A1; its continuation from this point is now the distal part or A2, which ascends in front of the lamina terminalis to pass into the longitudinal fissure between the hemispheres. The A2 segment is formed by the A2 (infracallosal), A3 (precallosal), A4 (supracallosal), and A5 (posterocallosal) segments [2]. The mean diameter of the AComA is 1.2 mm, and an AComA less than 1.0 mm is considered hypoplastic [3].

A rare variant that occurs in only 2-13.1% of individuals is the median artery of the corpus callosum (MACC) originating from the AComA [4]. When present, such a variant can be a surgical obstacle for neurosurgeons working in the suprasellar area, as well as alter cerebral hemodynamics and perfusion. Here, we report a duplicated MACC found during routine dissection of a cadaveric brain.

## Case presentation

**Figure 1:**
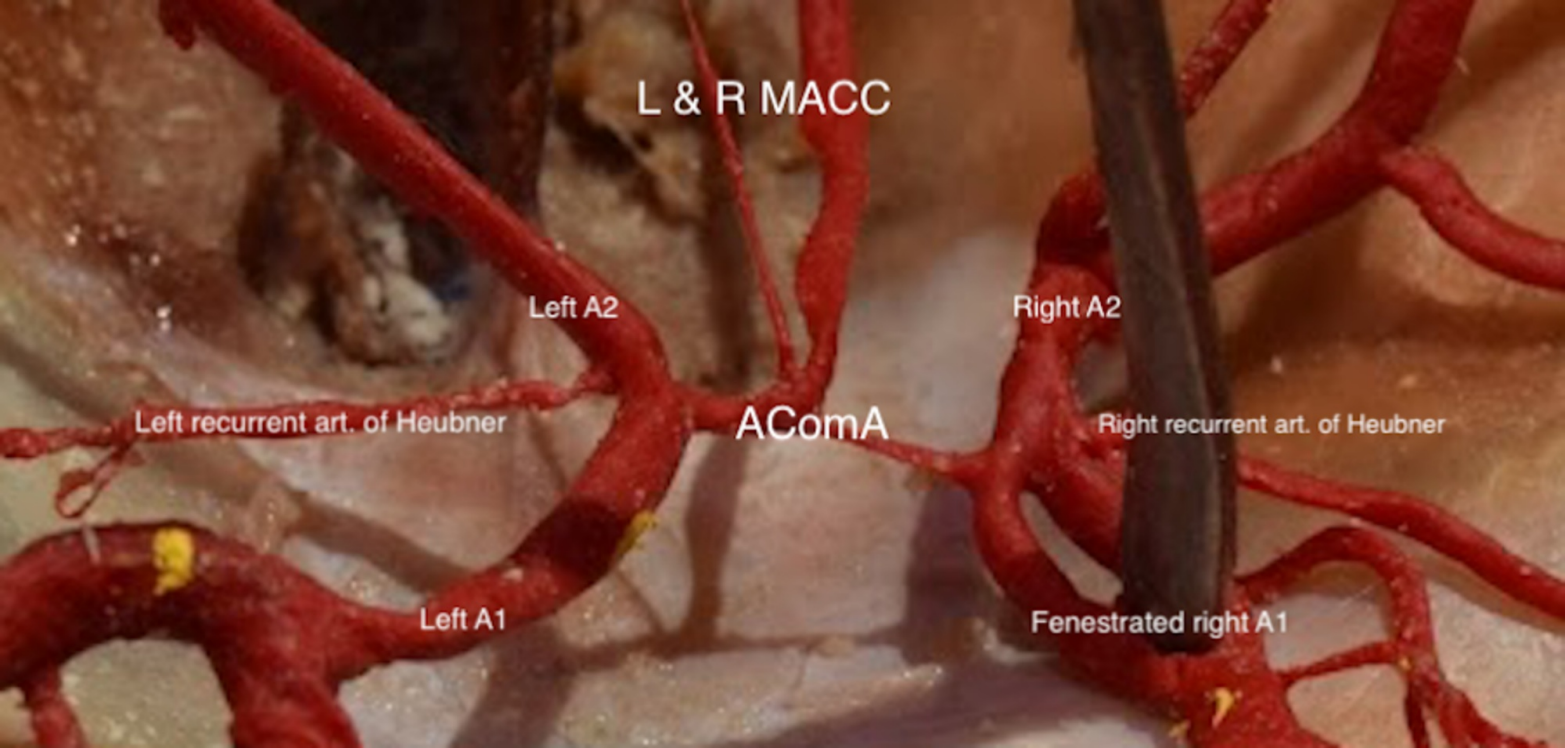
Anterior Communicating Artery Complex.

**Figure 2:**
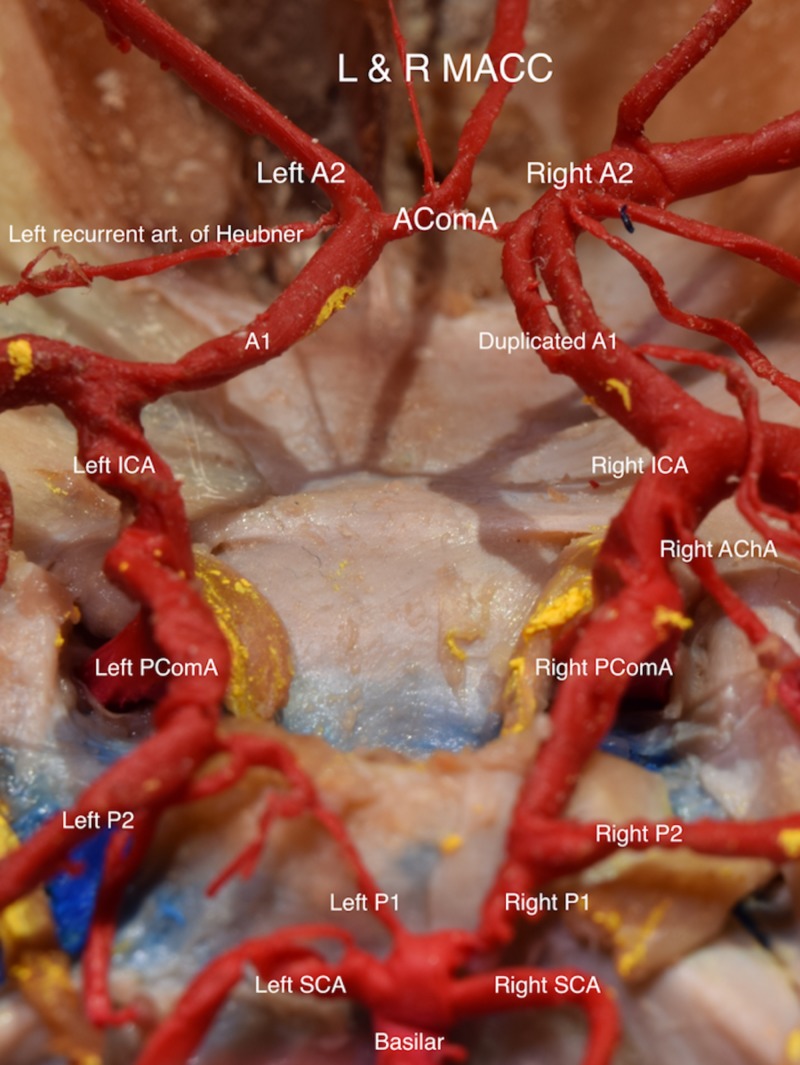
Circle of Willis.

During routine dissection and removal of cerebral structures of a 56-year-old female at death cadaver, duplicated branches of the AComA were observed. After further inspection, both arteries were found to originate from the anterior medial aspect of AComA (Figure 1). This was realized to be an extremely rare case of duplication of the MACC, which to our knowledge has never been reported before. The left MACC had a diameter of 0.60 mm and the right MACC had a diameter of 1.02 mm. The left A1 and A2 measured 1.96 mm and 1.74 mm, respectively, while the contralateral A1 was fenestrated measuring 1.06 mm and 1.07 mm joining into a common A2, which measured 2.04 mm. No hypoplasia was observed with the AComA although its course exhibited a variation in diameter of 1.01 mm (right side) and 1.13 mm (left side) (Figure 2). The left recurrent artery of Heubner measured 0.76 mm in diameter while the right one measured 0.80 mm in diameter. No other cerebral vascular variants or pathologies was observed.

## Discussion

Anatomically and physiologically speaking, the posterior communicating artery completes the circle of Willis by joining both vertebral arteries, and anteriorly the AComA serves a similar role by connecting both internal carotid arteries. Any anatomical variation in the course of this system may have hemodynamic repercussions for the patient and pose a surgical challenge if treatment is required [5].

The AComA complex consists of two anterior cerebral arteries (ACA), the AComA itself, and both recurrent arteries of Heubner (or medial striate branches). This complex has a strong clinical and surgical relevance since it is the most common site of intracranial aneurysms [6].

Under normal embryological development, the ACA has been identified at around 41-48 days of gestation. The embryo has already developed the primitive olfactory artery (POA), which by this date already has two branches, one to the nasal fossa and one running more medially to develop into the ACA. As development continues, the MACC (also termed the superior callosal artery, median callosal artery, accessory anterior cerebral artery, medial anterior cerebral artery, or the third A2 artery) appears to arise as a small embryonic branch of the AComA. When apoptotic signals fail to involute this artery, it then continues its course, runs parallel to the pericallosal artery, and supplies blood to the corpus callosum, septal nuclei, septum pellucidum, rostral portions of the fornix, and both frontal lobes [4, 7]. The incidence of this variant ranges from 2 to 13% with a female predominance [8]. As early as 1921, anatomical variants of the AComA complex have been considered to have direct relationships with the formation of aneurysms, with hypoplasia of the A1 segment as the most common variant, observed in approximately 80% of all cases of aneurysms in the AComA complex [9]. Of particular clinical importance regarding the MACC, it has been reported that aneurysms of the AComA use an MACC as their main draining artery [10], while Yanaka et al. have reported a case of ruptured aneurysms of the MACC at its origin [11]. Proper visualization of AComA is not easily achievable during cerebral imaging; orientation is not in a strict transverse plane as expected, but rather in an oblique or straight anterior-posterior fashion. Variation reporting the MACC using angi-MRI as the imaging method is around 3%, microsurgical observations while performing bifrontal craniotomies for clipping AComA aneurysms have found this number to be as high as 13.1% [12]. Confusion or misinterpretation of these anatomical scenarios may cause significant repercussions for the patient being treated as cases of occlusion of the MACC while treating AComA aneurysms have been reported [12, 13].

Structural peculiarities of the intracranial arteries that could lead to the formation of aneurysms when compared to the extracranial vessels include less elastic fibers in the tunica media and adventitia, less muscle in the media, thinner adventitia, and a more prominent elastic lamina. Minor resistance point hypothesis states that aneurysm formation occurs at a level where wall weakness is at its most, during flow direction changes, or during embryogenesis where primitive vessels have sprouted and may have later occluded or not, as in the case of MACC [14, 15]. Histological findings of the wall of aneurysms include disorganization of cellular components (muscle fibers, collagen type III and IV, etc.) with fragmentation or loss of the internal elastic lamina, intimal hyperplasia, loss of collagen I in the adventitia and of fibronectin in the media layer [14].

Treatment remains standard (endovascular versus surgical) depending on the aneurysm characteristics and location, and there have yet to be reported any follow-up differences regarding both treatments under this type of variation, whereas AComA aneurysms with an ACA trunk-dominant circle of Willis configuration (one A1 supplying both A2 while the other is hypoplasia) have shown less angiographic stability at follow-up than those with an ACA trunk-codominance similar to other “termination” type aneurysms [16].

## Conclusions

Although no other significant anatomical variant or vascular pathology was associated with this case report, it serves as an aid for further research in which this vascular anomaly may play a much more significant role in a setting of a higher clinical relevance such as when facing an AComA aneurysm. Inadequate visualization of the anterior communicating artery complex and its variations may lead to incorrect vessel identification resulting in catastrophic consequences for the patient. Cerebral hemodynamics and perfusion are also altered in the setting in which an MACC is present and this might lead to the formation of intracranial vascular pathologies.
